# Genomic Applications to Various Biological Questions

**DOI:** 10.2174/138920291505141106100438

**Published:** 2014-10

**Authors:** Rongling Wu

New biotechnologies have revolutionized the collection of various genome-wide (*i.e.*, omics) data across a range of organizational levels in a variety of species. By integrating these data with particular biological phenomena and processes, many questions of fundamental importance to agriculture, biology and biomedicine can be addressed. In this issue, a group of scientists provide creative applications of genomic approaches to solving several practical issues related to heat shock resistance, salt resistance, response to anticancer interventions, population genetic diversity and genome-wide association studies. Chen *et al. *identified genes from the leaf transcriptome whose expression is related to response to heat shock stress in *Populus euphratica*. Li *et al. *compared genomic differences in total levels of gene expression between two varieties of *Salix *in response

to salt stress, with the findings that can facilitate the precise selection of salt-resistant genotypes. Wang *et al. *used a powerful statistical model, called skellam, to study the genomic mechanisms that guide breast cancer cell lines to resist or sensitize to an anticancer drug. Zeng *et al. *developed a set of SSR markers to study the genetic diversity of outcrossing populations for *Carya cathayensis*, a hickory woody plant species naturally distributed in the southeastern China, and transferred these marker systems to other species in the same genus. Fu *et al. *proposed a graphical approach to correcting for multiplicity effect in genome-wide association studies.

The collection of articles published in this thematic issue present the uses of genomic approaches to tackle several biological issues. It is believed that genomic data through their integration, analysis and modeling will play a more and more important role in addressing the complexity of biological questions and elucidating the mechanisms of interactions between genes and phenotypic variation. In particular, current genomic approaches have provided a desirable opportunity to pursue an in-depth study of those underrepresented species, such as forest trees, highlighting their intrinsic importance to human beings.

